# Space Radiation and Plasma Effects on Satellites and Aviation: Quantities and Metrics for Tracking Performance of Space Weather Environment Models

**DOI:** 10.1029/2018SW002042

**Published:** 2019-10-15

**Authors:** Yihua Zheng, Natalia Yu Ganushkina, Pier Jiggens, Insoo Jun, Matthias Meier, Joseph I. Minow, T. Paul O'Brien, Dave Pitchford, Yuri Shprits, W. Kent Tobiska, Michael A. Xapsos, Timothy B. Guild, Joseph E. Mazur, Maria M. Kuznetsova

**Affiliations:** ^1^ Space Weather Laboratory NASA Goddard Space Flight Center Greenbelt MD USA; ^2^ Finnish Meteorological Institute Helsinki Finland; ^3^ Department of Atmospheric, Oceanic and Space Sciences University of Michigan Ann Arbor MI USA; ^4^ The Space Environment and Effects Section European Space Research and Technology Centre Noordwijk Netherlands; ^5^ Mission Environments Group, Jet Propulsion Laboratory California Institute of Technology Pasadena CA USA; ^6^ Institute of Aerospace Medicine German Aerospace Center Köln Germany; ^7^ NASA Langley Research Center Hampton VA USA; ^8^ Aerospace Corporation Chantilly VA USA; ^9^ SES Engineering Château de Betzdorf Luxembourg; ^10^ Helmholtz Centre Potsdam, GFZ German Research Centre for Geosciences Potsdam Germany; ^11^ Department of Earth, Planetary and Space Sciences University of California Los Angeles CA USA; ^12^ Space Environment Technologies Los Angeles CA USA; ^13^ Radiation Effects and Analysis Group NASA Goddard Space Flight Center Greenbelt MD USA

**Keywords:** space radiation and plasma effects on space assets, validation and metrics, space weather environment models, surface and internal charging, single‐event effects, radiation effects at aviation altitudes

## Abstract

The Community Coordinated Modeling Center has been leading community‐wide space science and space weather model validation projects for many years. These efforts have been broadened and extended via the newly launched International Forum for Space Weather Modeling Capabilities Assessment (https://ccmc.gsfc.nasa.gov/assessment/). Its objective is to track space weather models' progress and performance over time, a capability that is critically needed in space weather operations and different user communities in general. The Space Radiation and Plasma Effects Working Team of the aforementioned International Forum works on one of the many focused evaluation topics and deals with five different subtopics (https://ccmc.gsfc.nasa.gov/assessment/topics/radiation-all.php) and varieties of particle populations: Surface Charging from tens of eV to 50‐keV electrons and internal charging due to energetic electrons from hundreds keV to several MeVs. Single‐event effects from solar energetic particles and galactic cosmic rays (several MeV to TeV), total dose due to accumulation of doses from electrons (>100 keV) and protons (>1 MeV) in a broad energy range, and radiation effects from solar energetic particles and galactic cosmic rays at aviation altitudes. A unique aspect of the Space Radiation and Plasma Effects focus area is that it bridges the space environments, engineering, and user communities. The intent of the paper is to provide an overview of the current status and to suggest a guide for how to best validate space environment models for operational/engineering use, which includes selection of essential space environment and effect quantities and appropriate metrics.

## Key Points

- Providing an overview of the current status and proposing a guide for how to best validate space environment models for operational use
- Two types of physical quantities for both science and engineering purposes have been identified
- Proper metrics are needed for evaluating space environment models for different application purposes

## 1 Introduction

**Figure 1:**
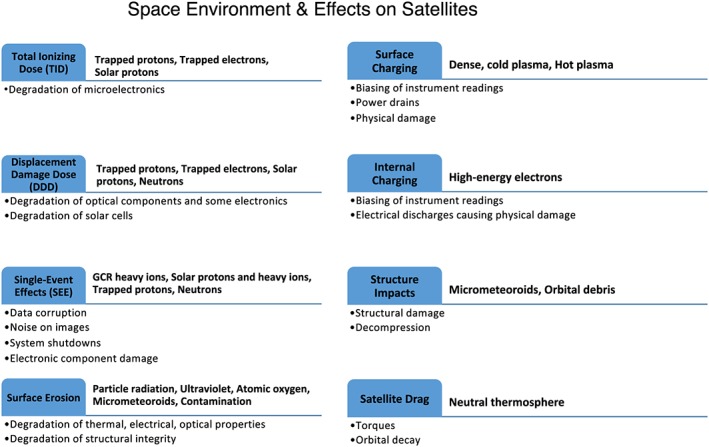
Summary of space weather impacts on satellites and their environment sources.

Space assets (including aircraft) are subject to an environment consisting of different particle populations that often evolve dynamically over time and space, and potentially bringing about deleterious effects on spacecraft electronics and/or life in space (e.g., Feynman & Gabriel, 2000). Figure 1 summarizes the main space weather impacts and their environmental sources, from a space hardware perspective. The blue boxes are used to show each impact with sources to its right and specific impacts under the blue line. Particles across a broad energy range contribute to satellite impacts, which include cold, dense, and hot electrons from a few eV to tens of keV that could lead to surface charging, energetic electrons that are above a few hundred keV possibly leading to internal charging, solar energetic particles (SEPs), galactic cosmic rays (GCRs), and trapped inner belt protons/ions that are sources for single‐event effects on spacecraft electronics (e.g., O'Bryan et al., 2009) and avionics (e.g., Dyer & Truscott, 1999; Normand, 1996). In addition, noncharged particles including UV radiation (photons), energetic neutrons, atomic oxygen, and neutral atmosphere could pose various hazards. Energetic protons, electrons, heavy ions, and neutrons can lead to total dose effects over time. Micrometeoroids and orbital debris are potential hazards for spacecraft as well. Table 12.1 in Daly et al. (2007) also provides a concise summary of space weather effects due to space environment.

GCR and SEPs can also have adverse effects on humans in space (e.g., Chancellor et al., 2014). From human perspective, space radiation can have acute in‐flight effects, long‐term cancer risks, and risks to the central nervous system and cardiovascular system.

The space environment and its associated effects span vast and complex domains and involve multiple disciplines such as space science, quantum physics, material science, biological and medical science (for human effects), and computational physics. Here we mainly focus on space environment specification, but with users' (types of users will be mentioned in section 3) needs in mind. Traditionally, space weather environment information (both models and observations) and engineering models of effects tend to exist in isolation and reside in different communities. To break the impasse and bridge the gaps and to make space environment models (primarily developed by scientists) more useful to the engineering and user community, it is imperative to have standardized and more user‐focused physical parameters/metrics to measure their performance over time, particularly the physical quantities that matter to engineers/users and that can be easily understood/translated in terms of impact assessment and monitoring. This serves as a key motivational force behind the International Forum for Space Weather Capabilities Assessment. It allows us to tackle problems related to space weather effects from one particular and tangible angle.

**Figure 2:**
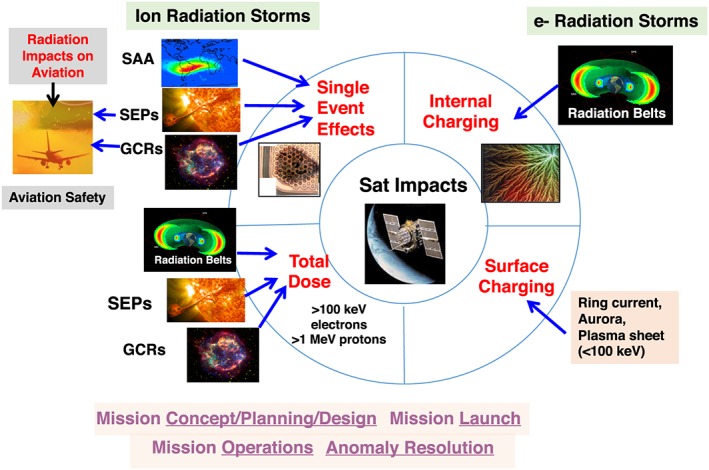
Space radiation and plasma impacts and their sources.

In cooperation with the community, the Space Radiation and Plasma Effects Working Team has been working together to select appropriate physical quantities/metrics that can be qualitatively translated into effects. It deals with how particles (mainly charged particles) at different energies affect satellites and airline passengers and hardware. The effects include surface charging, internal charging, single‐event effects (SEEs), total dose, and radiation effects at aviation altitudes. Figure 2 shows the focus/subdomains (the types of impacts and their sources) of the Space Radiation and Plasma Effects Working Team.

## 2 Space Radiation and Plasma Effects on Space Assets

### 2.1 Spacecraft Charging

Spacecraft charging (see NASA Handbooks, NASA‐HDBK‐4002A w/CHANGE 1, NASA‐HDBK‐4006) remains a serious operational threat for the design and operation of space assets. It usually manifests as surface charging and/or internal charging. When charge is built up either in the outside (surface) material or in the material (internal), an electrostatic discharge (ESD) can occur when the electric field exceeds the breakdown strength of the material. If the discharge occurred at or near a sensitive component, these ESD currents can cause compromised function and/or catastrophic destruction of sensitive electronics, solar array failures, uncommanded change in system states (phantom commands), loss of synchronization in timing circuits, spurious mode switching, power‐on resets, erroneous sensor signals, telemetry noise, and/or loss of data. Other concerns with discharges are possible electromagnetic interference and material damage. Electromagnetic interference can produce noise levels in receive bands that exceed the receiver sensitivity, communications issues due to the excess noise, or phantom commands or signals. ESDs can damage mission‐critical materials, including thermal control coatings, reentry thermal protection systems, and optical materials such as dielectric coatings and mirror surfaces. The re‐attracted photoionized outgassing materials can be deposited as surface contaminants. Surface and internal charging can also compromise science instrument and sensor functionality.

The distinction between surface charging and internal charging is that internal charging is caused by energetic particles that can penetrate and deposit charge very close to a victim site (e.g., Garrett, 2016, and references therein). Surface charging occurs on areas that can be seen and touched on the outside of a spacecraft. Surface discharges occur on or near the outer surface of a spacecraft and discharges must be coupled to an interior affected site rather than directly to the victim. Energy from surface arcs is attenuated by the coupling factors necessary to get to victims (most often inside the spacecraft) and, therefore, is less of a threat to electronics. External wiring and antenna feeds, of course, are susceptible to surface charging. Internal charging, by contrast, may cause a discharge directly to a victim pin or wire with very little attenuation if caused by electron deposition in circuit boards, wire insulation, or connector potting. It has been shown that differential charging followed by discharging is a major source of spacecraft anomalies (Koons et al., 2000).

Surface charging typically results from the buildup of charge on surfaces when assets are immersed in fluxes of charged particles. It also results from induced currents from asymmetric plasma flows or planetary magnetic fields (e.g., Garrett, 2016). The interaction of a spacecraft and a planetary ionosphere can generate a plasma wake (Ferguson, 1985; NASA‐HDBK‐4006, and references therein) that can distort the potentials around the vehicle, as demonstrated by the International Space Station. Additionally, electric fields caused by the movement of a conducting body across a planetary magnetic field can induce currents and result in charging in the structure. As indicated in Figure 2, ring current, aurora, and plasma sheet particles can be potential space environmental sources for surface charging (e.g., Ganushkina et al., 2017; Matéo‐Vélez et al., 2018).

Internal charging refers to the accumulation of electric charge on interior, ungrounded metals, or in the dielectrics inside a spacecraft by penetrating/energetic electrons. The resulting discharge is termed as Internal ElectroStatic Discharge, which may be even more common than originally thought (Bodeau, 2005, 2010).

A few eV to 50‐keV electrons are considered source for surface charging (Matéo‐Vélez et al., 2018) and electrons greater than 100 keV (>100 keV) are responsible for internal charging (the main source is radiation belt electrons). Sometimes it is difficult to differentiate between surface charging and internal charging as the root cause of an anomaly. The transitional energy of surface charging and internal charging is usually considered to be ~50–100 keV. Geosynchronous orbit (GEO) and its vicinity are believed to constitute one of the most susceptible regions for surface charging as electron injections from substorms or substorm‐like transients elevate the flux level of the electrons that are in the energy range for surface charging. In addition, auroral region and ring current are major sources as well. Charged particles for internal charging are mainly from the radiation belts.

For more details on spacecraft charging, its history, studies/understanding, and its mitigation techniques/practices, see the NASA Technical Handbooks regarding spacecraft charging (NASA‐HDBK‐4002A w/CHANGE 1, NASA‐HDBK‐4006; Ferguson & Hillard, 2003; Ferguson et al, 2015; Garrett, 2016, Frooninckx & Sojka, 1992; and references therein).

### 2.2 Space Radiation Effects on Spacecraft Electronics

Space radiation environment consists of SEPs, GCRs, energetic particles trapped in the South Atlantic anomaly region, and energetic electrons in radiation belts. Radiation effects on electronics can be classified into two classes: those caused by the total accumulated radiation dose over the life of a mission (gradual) and those caused by single‐event effects (sudden/transient). In general, the basic effect of radiation‐matter interaction is to bring energy deposition into the target object. Depending on the particle species and energy, and physical processes involved in the targeted material/structure, this energy deposition will result in a variety of effects.

#### 2.2.1 SEEs

SEEs are a serious problem for electronics operated in space (e.g., Edmonds, Barnes, and Scheick, 2000—a JPL publication; O'Bryan et al., 2009; Xapsos et al., 2007), and they are becoming an issue for advanced technologies in avionics (e. g., Dyer & Truscott, 1999; Dyer et al., 2018), and even at sea level. The charge deposited by a single ionizing particle (producing a dense track of electron‐hole pairs in devices, circuits, and components) can produce a wide range of effects, including single‐event upset (transient and nondestructive, affecting mainly memories), multiple bit upset (nondestructive), single‐event transient (nondestructive), single‐event functional interrupt (nondestructive), single‐event latch‐up (destructive, affecting mainly complementary metal‐oxide‐semiconductor structure), single‐event burnout (destructive; affecting mainly power metal‐oxide‐semiconductor field‐effect transistors), single‐event gate rupture (potentially destructive, affecting mainly submicronic structure), and single hard error (another destructive effect). In general, the sensitivity of a technology to SEE increases as the device dimension decreases and as the circuit speed increases.

#### 2.2.2 Total Dose Including Total Ionizing Dose and Displacement Damage Dose

When a charged particle (or a photon) travels through a material, it interacts with electrons in the material and causes some of the atoms to become ionized, creating electron‐hole pairs. Such effects accumulate in insulators (e.g., a gate oxide in a complementary metal‐oxide‐semiconductor device). The accumulated trapped charge is measured by the accumulated ionization, which in turn is measured by the sum (over particles) of the energy lost by the particles to the material via interactions with the electrons. Therefore, a useful measure is the total energy, per unit mass of material, transferred to the material via ionization from all ionizing particles, which is called the total ionizing dose (TID; e.g., Cochran et al., 2009; Edmonds et al., 2000).

The TID, mostly due to electrons and protons, can result in device failure (or biological damage to astronauts). In either case, TID can be measured in terms of the absorbed dose, which is a measure of the energy absorbed by matter. Absorbed dose is quantified using either a unit called the rad (an acronym for radiation absorbed dose; 1 rad = 100 ergs/g) or the SI unit which is the gray (Gy): 1 Gy = 100 rads = 1 J/kg (J: joule, kg: kilogram).

The TID is calculated from the trapped protons and electrons, secondary Bremsstrahlung photons, and solar energetic particles (the contribution from galactic cosmic ray ions is negligible in the presence of these other sources). The “dose profile curve” that indicates the dose received through a shield of varying thickness (most often a hollow aluminum sphere) is usually used for evaluating the TID on a component.

Displacement damage (e.g., Edmonds et al., 2000; Jun et al., 2003) is the result of nuclear interactions, typically scattering, which cause lattice defects. Displacement damage is due to cumulative long‐term nonionizing damage from protons, electrons, and neutrons. The collision between an incoming particle and the nucleus of a lattice atom subsequently displaces the atom from its original lattice position.

The particles producing displacement damage include protons of all energies, electrons with energies above 150 keV, and neutrons (e.g., from onboard power sources). Shielding has some effect, but it depends on location of the device (e.g., solar cells). Displacement damage is typically of lesser concern than single‐event effects or TID, although protons cause displacement damage in solar cells (Messenger et al., 2001, 2004, 2014) and bipolar devices. Displacement damage degrades minority carrier lifetime; a typical effect would be degradation of gain and leakage current in bipolar transistors.

The total energy loss per unit distance of travel is called the linear energy transfer, or LET. The LET is usually normalized by dividing by the density of the medium; the most popular units are MeV‐cm^2^/mg. The reason for this normalization is that it makes the LET for a given particle and energy similar in different materials. LET depends also on the incident particle species and energy.

The interactions of radiation particles with materials and resulting effects on different types of devices and electronic components are very complex (e.g., Cochran et al., 2009; O'Bryan et al., 2009). More details can be found in various publications (e.g., Edmonds et al., 2000; Srour & McGarrity, 1988; Velazco et al., 2007).

### 2.3 Space Radiation Effects at Aviation Altitudes

The primary source of radiation hazards at aviation altitudes are from GCRs and SEPs.

**Figure 3:**
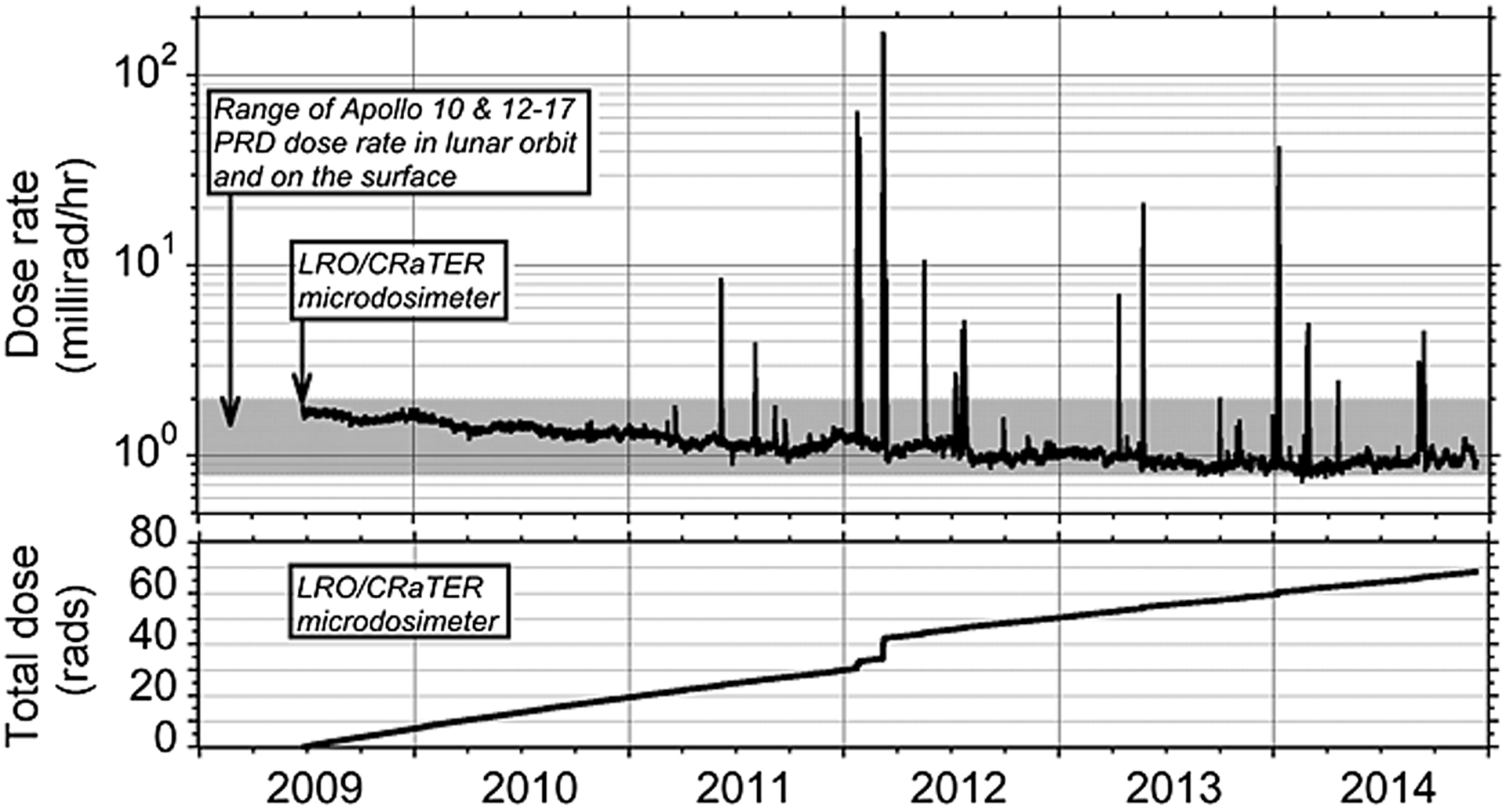
LRO (Lunar Reconnaissance Orbiter)/CRaTER (Cosmic Ray Telescope for the Effects of Radiation) microdosimeter measurements from launch in June 2009 to December 2014. Doses due to SEPs appear as spikes while those from GCRs is the slowly varying background (Mazur et al., 2015; Figure 1).

GCRs have energies (10^8^–10^20^ eV/nucleon) much higher than SEPs (10^6^–10^10^ eV/nucleon). GCR ions are typically GeV (gigaelectron volt) and above while SEP ions are in the energy range of tens MeV to hundreds MeV. For some extreme SEP events, ions can be accelerated to GeVs and higher. In the near‐Earth's environment and (within the heliosphere in general), GCR flux (dose) is at continuous background levels while SEP fluxes/dose are highly dynamic and can vary several orders of magnitude (“spikes” in Figure 3) on a short time scale. During large SEP events the intensity of >100‐MeV protons hitting the upper atmosphere can be >1,000 times that of GCR protons. Other speculative radiation sources affecting aviation (if present) might be precipitating energetic electrons from the radiation belt as discussed by Tobiska et al. (2016) and Terrestrial Gamma ray Flashes. Further measurements and analyses need to be done to ascertain their contribution to the atmospheric radiation environment. While ionizing radiation from GCRs and SEPs pose health consequences/risks (such as long‐term cancer risks and potential damage to the central nervous system and cardiovascular systems) to airline passengers and crews (human in space in general), SEEs on avionics from high‐energy particles and low‐energy, thermalized neutrons (via their interactions with nuclei inside avionic systems) are also concerns for aviation (e.g., Dyer & Truscott, 1999; Normand, 1996; Tobiska et al., 2015). However, the working team's initial efforts in this area have been mainly on the radiation effects in terms of dose or dose equivalent on passengers and aircrews. SEEs on avionics will be part of future expertise and model expansion. Such impacts can be combined into SEEs on space hardware/systems in general, although SEEs on avionics require accurate understanding and modeling of the particle and atmosphere interactions, together with magnetic rigidity cutoff consideration.

## 3 Users

The working team has identified potential user groups. For surface charging, internal charging, single‐event effects, and total dose, the users are more or less similar.

1. Satellite designers for both commercial and government
2. Satellite operators and anomaly analysts for both commercial and government
3. Scientists for both academia and government
4. Insurance companies

For radiation effects at aviation altitudes, the users are mainly air crews, passengers, regulators, airlines, and scientists studying the relevant environment.

## 4 Physical Quantities and Metrics

### 4.1 Physical Quantities

Table 1 shows the physical parameters that have been selected from both engineering and science perspectives, following team discussions. The science quantities are carefully chosen so that through a unified and agreed‐upon engineering effect model (with a simplified geometry, default material, and so on), the impact can be readily computed/assessed, though may be qualitative due to the complexities and lack of a thorough understanding and testing associated with impact analysis. For example, NASCAP‐2K can be used for surface charging, DICTAT/NUMIT for internal charging, SHIELDOSE‐2 for total dose, CRÈME 96 for single‐event effects, and NAIRAS for radiation effects on aviation. More information (including references) about the models mentioned here can be found in section 7. The effect quantities have been found to correlate with each type of observed effects/anomalies (e.g., O'Brien, 2009; Thomsen et al., 2013; Wrenn & Smith, 1996; Wrenn et al., 2002; Edmonds, Barnes and Scheick, 2000—a JPL publication; NASA‐HDBK‐4002A, NASA‐HDBK‐4006). In addition, the time scale relevant to the effects is noted in the last column. For example, the integral flux of the greater than 10‐keV electrons is correlated well with surface charging anomalies. From science perspective, the same quantity plus electron temperature and density will be examined for model and data comparison. In contrast, internal charging is an accumulative effect over a certain time period, such as a 24‐, 48‐, and 72‐hr interval or even longer. The 100 fA/cm^2^ [100 mils] (meaning 100 femtoampere/cm^2^ behind 100 mils aluminum shielding; 1 mil = 0.001 in. = 25.4 μm) is a threshold for internal charging problems and will be used as an engineering quantity for analyzing internal charging effects. Energetic electron flux at 1 MeV or the integral flux at greater than 2 MeV have been selected from the science perspective. A point to note is that 100 mils is the nominal aluminum shielding thickness and 4 mils (~100 μm) is the nominal cover glass thickness for solar cells onboard GEO spacecraft using chemical propulsion orbit raising. Nowadays, Electric Orbit Raising is increasingly used for GEO missions (e.g., Horne & Pitchford, 2015) and the extended period spent in the inner radiation belt means that thicker cover glasses—150 to 200 μm (6 to 8 mils) are becoming common. And for spacecraft operating in low Medium Earth Orbit (such as SES's O3b constellation), much thicker cover glasses are often used (typically 600–800 μm).

**Table 1:** The Physical Quantities Chosen for Validation From Both Science and Engineering Perspectives

	Effect quantity	Science predictands	Time scale (Space Weather)
Surface charging	>10‐keV electron flux	>10 keV e− flux, Te, Ne	Seconds
Internal charging	>100 fA/cm^2^ [100 mils]	1 MeV and >2 MeV e− flux	24‐hr averaged
Single‐event effects	SEE rate [100 mils]	>30 MeV p+ flux, >15 MeV·cm^2^·mg^−1^ LET flux	5 min, daily, weekly
Total dose	Dose in Si [100 mils, 4 mils]	30–50 MeV p+ flux, >1.5 MeV e− flux, 1–10 MeV p+	Daily, weekly
Atmospheric radiation	Dose rate in aircraft (*D* index)	Two spectral parameters (power law with rigidity)	5 min, hourly

For SEEs, the science quantities for consideration are the >30‐MeV proton flux or the >15‐MeV‐cm^2^/mg LET flux (as discussed above, LET has the advantage in that for a given particle and energy, the LET value is **nearly** the same in different materials. However, it is not a perfect quantity for space weather modelers as they need to take another model to transport flux through shielding and then calculate LET. It may be replaced by the >100‐MeV/nucleon heavy ion flux). SEE rate behind 100‐mils aluminum spherical shielding is used as an engineering quantity. Temporal scales of interest are 5 min, daily, or weekly. It should be noted that SEE rate is energy, composition/species dependent (heavy ions pose greater concerns, yet observationally their measurements are not readily available), and device dependent. For total dose effects, the 30–50‐MeV proton flux, the greater than 1.5‐MeV electron flux, and the 1–10‐MeV proton flux are the science quantities for evaluation of the environment models. The dose in silicon behind different levels of shielding such as 100 mils, 4 mils is the quantity for assessing the impact. Since total dose is an accumulated long‐term effect, the time scales of interest are daily, weekly, yearly, or mission lifetime. For radiation effects at aviation altitude, geomagnetic shielding, atmospheric shielding, and the influence of the solar wind need to be considered. Dose rate or dose equivalent rate (e.g., the rates of the ambient dose equivalent and the absorbed dose in silicon are used in Meier et al. (2018)) in aircraft is used for assessing the impact. For effective communication with users in the aviation community, the *D* index, which is directly based on dose rates by solar energetic particles in the atmosphere, has been suggested (Meier & Matthiä et al., 2014, 2018) instead of the *S* scales (https://www.swpc.noaa.gov/noaa-scales-explanation) that are based on particle fluxes with energies above 10 MeV outside the atmosphere. Although the *S* scales have been used by National Oceanic and Atmospheric Administration/Space Weather Prediction Center for classification of the effects of solar radiation storms (SEP events) on different infrastructures, their use for the radiation assessment at aviation altitudes is rather limited due to the fact that the threshold value 10 MeV (for the integral flux) is far too low for causing significant radiation exposure deep in the atmosphere at flight altitudes. The *D* index has been used as an operational quantity to inform airlines in Germany since 2014. Furthermore, it is also used by the U.S. Federal Aviation Administration operating Maps of Ionizing Radiation in the Atmosphere, the latest upgrade of its Solar Radiation Alert System, providing near‐real‐time (lag of 5–10 min) calculations of dose rates in the atmosphere during solar proton events through the U.S. National Oceanic and Atmospheric Administration's Weather Wire Service, which is publicly accessible (Copeland, 2018; Copeland et al., 2018).

Understandably, there are great complexities involved in assessing engineering effects (such as different material dependencies and geometry), compounded by our still insufficient knowledge/lack of understanding of the space environment's interactions with spacecraft (e.g., Hands et al., 2018) and inadequate testing. The situation is even worse for quantifying impacts on humans. However, the quantities that have been carefully selected in Table 1 should be able to serve as the starting set for tracking performance of space environment models over time. Much like the 500‐mb constant pressure charts used by meteorologists, here we are trying to find the key parameters that can provide a quick glimpse of potential engineering effects. Identifying the right parameters/quantities for corresponding space weather hazards is the crucial first step, as pointed out by Feynman and Gabriel (2000).

One of the commonalities among the five types of different effects is that energy spectrum is needed for an accurate assessment of the corresponding effects. In terms of energy spectra, power law distributions are quite common and important for our understanding of natural and man‐made phenomena. For power law distribution in energy, it is often difficult to measure the tail end of the distribution (at very high energies; e.g., Clauset et al., 2009). Also, the particles and plasmas relevant in this paper cover a wide range of energies and exist in different regions of space; besides the power law energy spectra, there are other varieties such as double power law (Mewaldt, 2006), relativistic kappa‐like distribution (Xiao et al., 2008), and bump on tail (Zhao et al., 2017). How different types of energy spectra affect the validation results is beyond the scope of this paper.

### 4.2 Metrics

Different types of metrics will be used to evaluate model performance. We will explore both the traditional and relatively new ones (details can be found below). In addition, through collaboration, we will also leverage the terrestrial weather forecast verification and model evaluation tools, such as the National Center for Atmospheric Research's Model Evaluation Tool (MET) for our extended model verification and validation efforts. Generally speaking, the types of metrics chosen should reflect, and be relevant to, the types of applications. The ultimate goal is to identify the metrics that matter most, which is expected to be an evolving and iterative process.

We will start with the common ones where they are relevant.

#### 4.2.1 Traditional Metrics

##### 4.2.1.1 Root‐Mean‐Square Difference

One of the most meaningful and widely used ways to evaluate model performance is to calculate root‐mean‐square difference between the model estimates and observations defined as

$$\;\mathrm{RMS}=\sqrt{\frac{\sum {\left(x_{\mathrm{obs}}-x_{\mathrm{mod}}\right)}^{2}}{N}}$$

where *x*_*obs*_ and *x*_*mod*_ are the observed and modeled values, respectively. Root‐mean‐square (RMS) difference has the same unit as observed and modeled values, *x*_*obs*_ and *x*_*mod*_. Perfect model predictions have RMS differences of 0. Therefore, the closer the RMS error is to 0, the more accurate the model is.

##### 4.2.1.2 Prediction Efficiency

Prediction efficiency, one of the skill scores against the mean of observations, is also commonly used to describe performance of models:

$$\;\mathrm{PE}=1-\frac{{\mathrm{RMS}}_{\mathrm{mod}}}{{\mathrm{RMS}}_{\mathrm{ref}}}=1-\sqrt{\frac{\sum {\left(x_{\mathrm{obs}}-x_{\mathrm{mod}}\right)}^{2}/N}{\sum {\left(x_{\mathrm{obs}}-<x_{\mathrm{obs}}>\right)}^{2}/N}}$$

where *x*_*obs*_ and *x*_*mod*_ are again the observed and modeled values and <*x*_*obs*_> is the mean value of the observed measurements. In this study, the mean value of the observations <*x*_*obs*_> was considered as a reference model instead of any empirical model. The prediction efficiency ranges from negative infinity to 1. A prediction efficiency of 1 implies a perfect model performance, while a prediction efficiency of 0 means that the model performance is as accurate as the mean of the observed data. A negative value indicates that the observed mean is a better predictor than the model.

##### 4.2.1.3 Ratio of the Maximum Change in Magnitudes and Ratio of the Maximum Magnitudes

The root‐mean‐square error and prediction efficiency measure how well time series observed data and modeled values are correlated with each other. Metrics based on ratio are used to quantify the model capability to produce peak values or short‐term variations during a certain period of time, even though performance of model is poor in term of the RMS error and/or prediction efficiency. The two types of ratio selected were the ratio of the maximum change (maximum minus minimum values; max − min, also called prediction yield) and the ratio of the maximum (max) values of models to those of observations during a certain time interval:

$$\;\text{ratio}\left(\max-\min\right)=\frac{{\left(x_{\mathrm{mod}}\right)}_{\max}-{\left(x_{\mathrm{mod}}\right)}_{\min}}{{\left(x_{\mathrm{obs}}\right)}_{\max}-{\left(x_{\mathrm{obs}}\right)}_{\min}},$$

$$\;\text{ratio}\left(\max\right)=\frac{{\left(x_{\mathrm{mod}}\right)}_{\max}}{{\left(x_{\mathrm{obs}}\right)}_{\max}}$$

where (*x*_*obs*_)_*max*_ and (*x*_*mod*_)_*max*_ are the maximum values of the observed and modeled quantities during a certain time window. Perfect models have a ratio of 1. The ratio of max‐min and the ratio of max larger than 1 overestimate maximum variations and maximum values. Note that the two ratios depend on the length of time window.

##### 4.2.1.4 Ratio of the Event (or Over a Certain Duration) Sum

$$\;\text{ratio}\left(\mathrm{sum}\right)=\frac{\sum x_{\mathrm{mod}}}{\sum x_{\mathrm{obs}}}$$

Such metrics may be used for comparing total accumulated dose type of quantities, say the dose over a flight.

##### 4.2.1.5 Relative Deviation and Mean Deviation

As used in Meier et al. (2018), the relative deviation of observed quantity to the modeled one can be defined as follows:

$$\;\Delta_{i}=\frac{x_{i}^{\text{model}}-x_{i}^{\text{meas}}}{x_{i}^{\text{meas}}}$$

The mean deviation Δ for a given event/interval (with n measurements) can be defined as

$$\;\bar{\Delta }=\frac{\sum_{i}^{n}|\Delta_{i}|}{n}$$

##### 4.2.1.6 Correlation Coefficient

It is a numerical measure of a statistical relationship between two variables. The Pearson correlation coefficient, *r*, is often used, defined as the covariance of the variables divided by the product of their standard deviations.

$$r=\frac{\sum_{i=1}^{n}\left(x_{\mathrm{obs}¯i}-\bar{x_{\mathrm{obs}}}\right)\left(x_{\mathrm{mod}¯i}-\bar{x_{\mathrm{mod}}}\right)}{\sqrt{\sum_{i=1}^{n}{\left(x_{\mathrm{obs}¯i}-\bar{x_{\mathrm{obs}}}\right)}^{2}}\sqrt{\sum_{i=1}^{n}{\left(x_{\mathrm{mod}¯i}-\bar{x_{\mathrm{mod}}}\right)}^{2}}}$$

*r* can take a range of values from +1 to −1, with 0 indicating that there is no association, a value greater than 0 indicating a positive association, and a value less than 0 indicating a negative association.

It should be mentioned that for flux (data that cover orders of magnitude) type of model and data comparison, the metrics above should be performed after applying the logarithmic calculation. For dose type of quantities, there is no such need.

##### 4.2.1.7 Categorical Skill Scores

- Threshold based (yes/no prediction)
  For example, for surface charging, whether the >10‐keV flux exceeds a certain threshold 1.5 × 10^7^ (1/cm^2^/s/str).

1. Heidke Skill Score (HSS)
  This is suitable when there are many events.
  The HSS calls for generation of a contingency table of hit (H), miss (M), false positive (F), and correct negative (N) model predictions. Their definition is as follows.
  ○ Hit: both observation and model exceed the threshold at least once in a time window
  ○ Miss: observation exceeds the threshold but model does not exceed threshold at least once in a window
  ○ False positive: model does exceed threshold at least once in a window, but observation does not
  ○ Correct negative: both observation and model do not exceed threshold in a window
  Skills:
  ○ Probability of detection: H/(H + M)
  ○ Probability of false detection: F/(F + N)
  ○ Heidke Skill Score: HSS = 2(HN – MF)/[(H + M) (M + N) + (H + F) (F + N)]
  The HSS measures the fractional improvement of the forecast over the standard forecast. Like most skill scores, it is normalized by the total range of possible improvement over the standard, which means that Heidke Skill scores can safely be compared on different data sets. The range of the HSS is –∞ to 1. Negative values indicate that the chance forecast is better, 0 means no skill, and a perfect forecast obtains a HSS of 1. The HSS is a popular score, partly because it is relatively easy to compute and perhaps also because the standard forecast, chance, is relatively easy to beat.

For example, Ganushkina et al. (2015, 2019) used HSS (it is called binary event tables/analysis there) for evaluating the performance of their nowcast model for low‐energy electrons in the inner magnetosphere that could constitute surface charging risks.

#### 4.2.2 Novel Metrics

##### 4.2.2.1 Metrics Based on the Log Accuracy Ratio

In Morley et al. (2018), metrics based on the log accuracy ratio have been suggested. Two useful ones are the Median Symmetric Accuracy and the Symmetric Signed Percentage Bias (SSPB). The advantages of them include the following: (1) they are meaningful for data spanning several orders of magnitude, (2) underprediction and overprediction by the same factor are penalized equally, (3) they are easy to interpret, and (4) they are robust to the presence of outliers and bad data.

###### 4.2.2.1.1 The Median Symmetric Accuracy

$$\varsigma =100(\exp\left(M\left(\left|\log_{e}Q_{i}\right|\right)-1\right)$$

where $\;Q_{i}=\frac{x_{\mathrm{mod}}}{x_{\mathrm{obs}}}$ is the ratio of predicted versus observed, *x*_*mod*_ is the model, *x*_*obs*_ is the observation, and M is the median value.

The median symmetric accuracy (ζ) is equivalent to the median percentage error.

###### 4.2.2.1.2 The SSPB

$$\;\text{SSPB}=100\;\mathrm{sgn}\;\left(M\;\left(\log_{e}\left(Q_{i}\right)\right)\right)\left(\exp\left(\left|M\left(\log_{e}\left(Q_{i}\right)\right)\right|\right)-1\right)$$

where Sgn is the signum function and M is the median value.

The SSPB can therefore be interpreted similarly to a mean percentage error but is not affected by the likely asymmetry in the distribution of percentage error and robustly estimates the central tendency of the error.

##### 4.2.2.2 Statistical Metrics

Given the chosen environmental quantity, the 75th and 97th percentiles (or other values) can be selected as the threshold values for defining green and red (hazard indicators) type of risks (by these definitions, the environment is green 75% of the time, yellow 22% of the time, and red 3% of the time). Computing the percentile value for both the observed and modeled quantity and examining their difference are the required steps. This type of metrics assesses both observed and modeled quantity's statistical significance in their entire distribution space/time. This type of metrics validates the modeled quantities' role from a long‐term, mission‐averaged perspective (its current percentile over a long period of time, whether it is in a green, yellow, or red zone), not just to validate a flux value, dose rate, or induced current. Its principle is similar to what is done in O'Brien (2009). Such metrics is not likely to be a first choice as it requires data of a long time period and running a model over the same long period correspondingly.

#### 4.2.3 Selecting Proper Metrics

Which metrics to use depends on the physical quantities (whether varying over several orders of magnitude or not) and types of models. However, for models of similar nature, same sets of metrics should be applied and compared. For example, the initial model validation work (Yu et al., 2019) on surface charging used the 10–50‐keV electron flux as the quantity for comparison. Different types of metrics were employed to evaluate model performance, including cross‐correlation, prediction efficiency, root‐mean‐square error, prediction yield, and the symmetric signed percentage bias but all were done to the **logarithmic value** of fluxes. In contrast, the initial validation work (Meier et al., 2018) of aviation radiation models used the simple relative deviation in the ambient dose equivalent rate dH*(10)/dt and in the absorbed dose rate in silicon *dD*_*Si*_/dt. The outcome/measure of metrics also depends on other factors such as boundary conditions and whether additional data are used in a particular model. All factors should be kept in mind for the fairness of the validation results. The CAMEL (Comprehensive Assessment of Models and Events based on Library tools) system to be discussed next (section 5) will provide choices of different metrics (could be one or more) that are suitable for quantities/models at consideration (Rastätter et al., 2019). Besides metrics evaluation for individual events, evaluation of a model performance over multiple events or an extended time interval will also be carried out. Statistical significance of different metrics will depend on the duration of an individual event or whether metrics itself is defined/based on many events (such as Heidke Skill Score or Statistical Metrics mentioned above).

The example below demonstrates that the choice of appropriate metrics depends on the chosen physical quantities or applications at hand.

**Figure 4:**
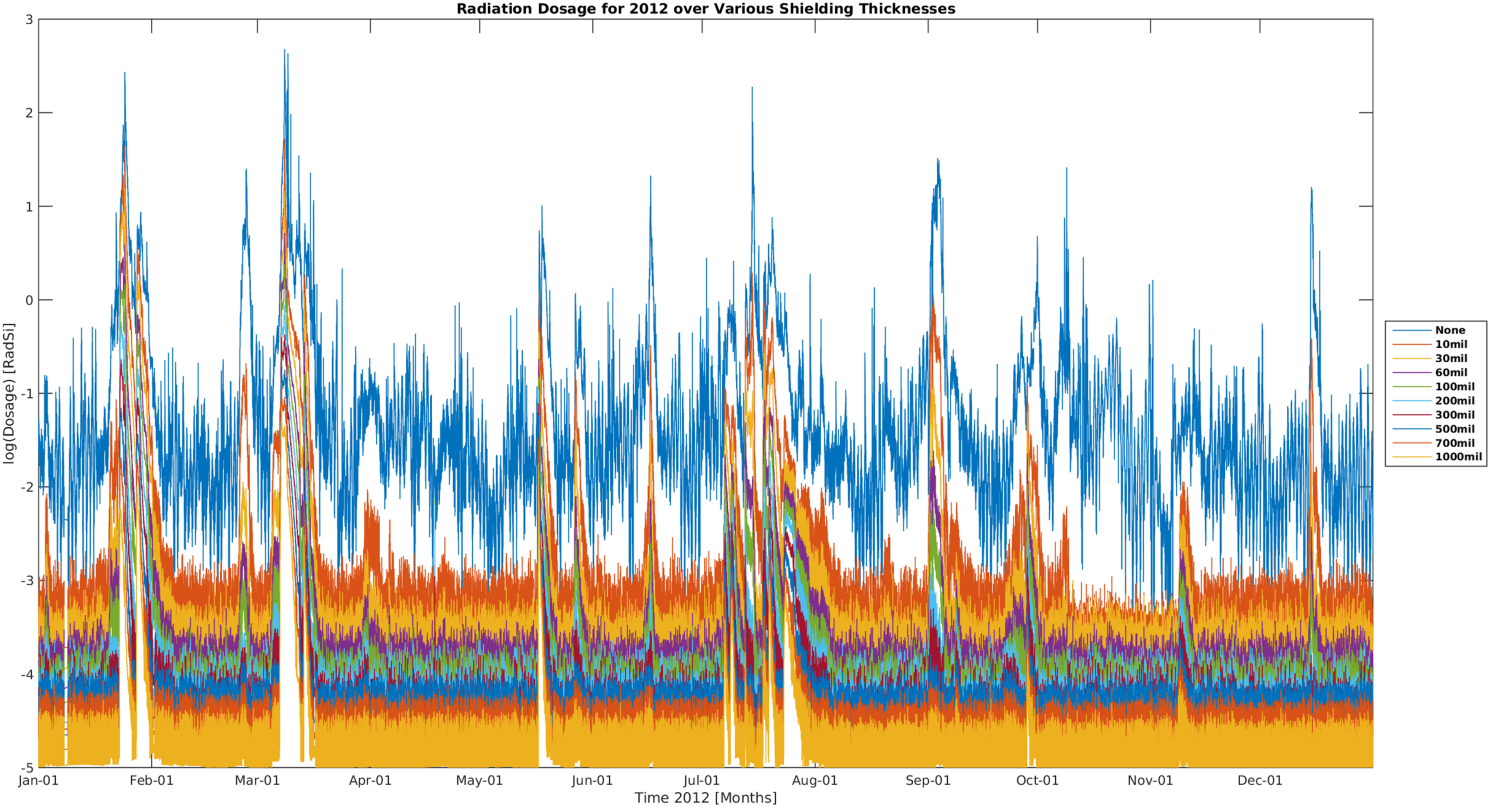
The absorbed dose in silicon for aluminum shielding of different thicknesses (image credit: Jean‐Paul Breuer).

Figure 4 shows the absorbed dose in silicon computed from the Numerical Optimizations, Visualizations, and Integrations on Computer Aided Design/Constructive Solid Geometry Edifices (also known as NOVICE) model (Jordan, 1976) for different thicknesses (indicated by different colors) of aluminum shielding for the year 2012. Particle spectra used in the NOVICE model are taken from GOES measurements. From the plot, we can see clearly the episodic nature of several SEP events during the year.

**Figure 5:**
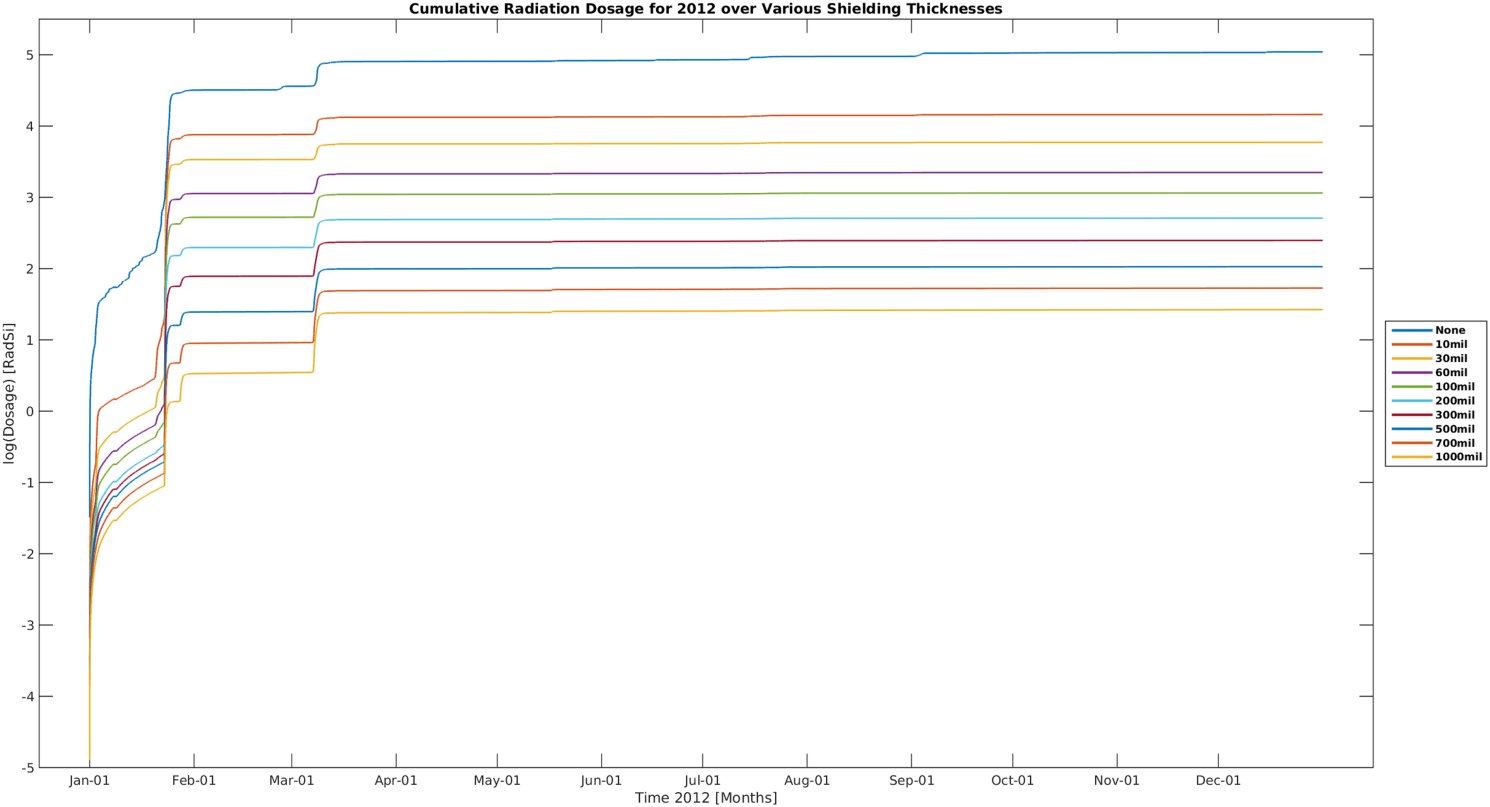
The accumulated dose profile in silicon for different level of aluminum shielding (image credit: Jean‐Paul Breuer).

Figure 5 shows the accumulated dose for the same year for different levels of shielding using the same GOES spectra data. We can see that the accumulated dose profile does not change much after the major SEP events in January and March of 2012.

Obviously, metrics suitable for model validation efforts for doses of individual events in Figure 4 and for the accumulated dose over a long time period in Figure 5 will be rather different with the former calling for “median symmetric accuracy” type of metrics and the latter calling for “mean deviation” type ones. Additionally, to reflect a model's performance from different perspectives, different metrics should be explored. For example, one model on SEPs that captures well the thin‐shielding situations may not perform well for thick shielding. Similarly, a model may perform well in terms of capturing the high‐energy tail but may suffer at the lower energy end.

## 5 Community Coordinated Modeling Center Resource: CAMEL

One resource relevant to model validation is the CAMEL system that has been under development. It is a framework to combine tools to perform model execution, postprocessing, and model evaluation. For details, please see the CAMEL paper of this special issue (Rastätter et al., 2019). This tool stores model outputs and observations for all validation studies, plots model and observations together, has built‐in variety of metrics, and is to incorporate features of the National Center for Atmospheric Research's Model Evaluation Tool (MET) through partnership with Tara Jensen, Barb Brown et al. (Jensen & Brown, 2018). MET is a verification toolkit designed for flexible yet systematic evaluation for terrestrial weather forecast.

## 6 NASA Standard for Models and Simulations

**Figure 6:**
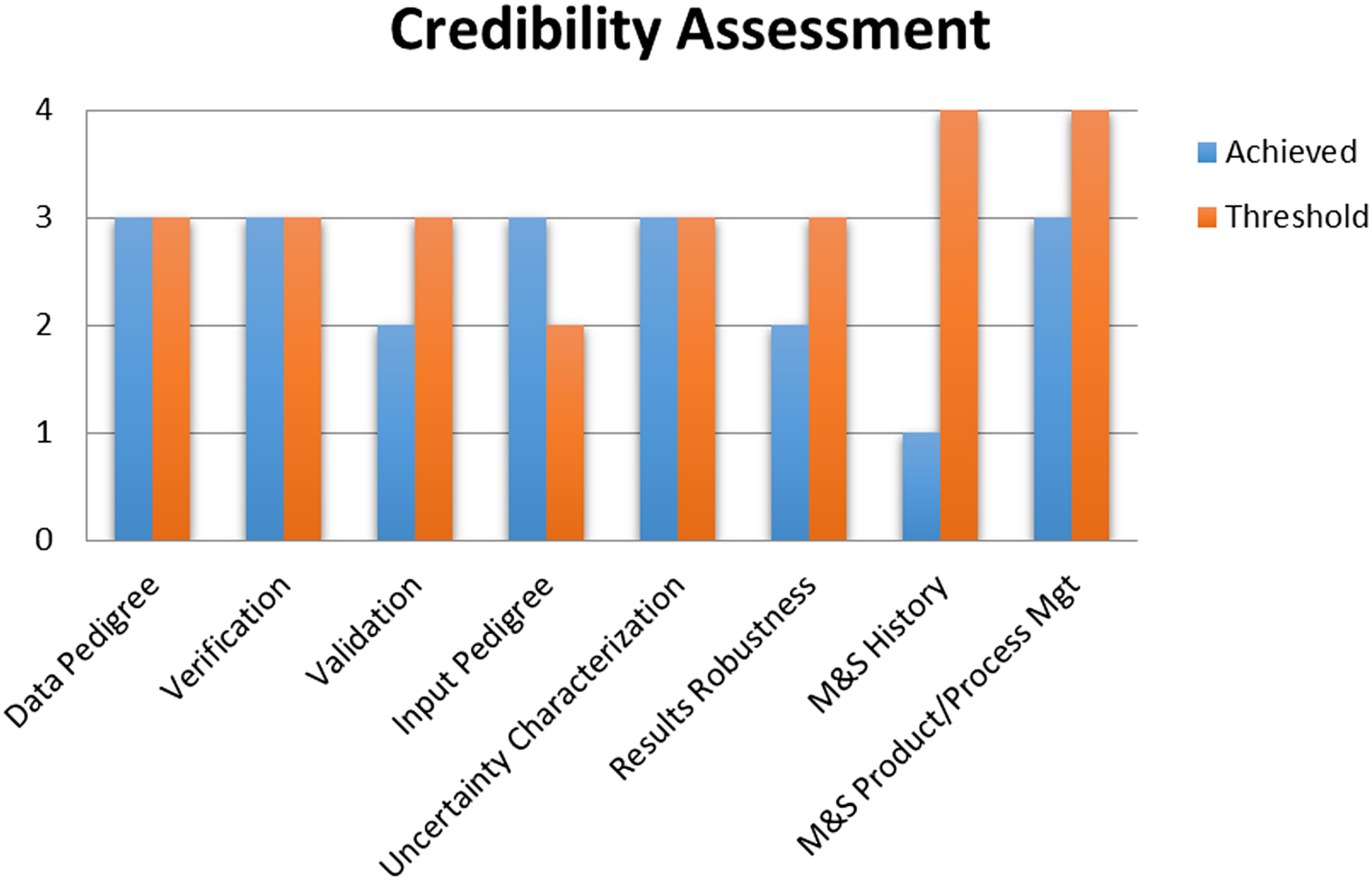
Bar graph of credibility assessment.

**Figure 7:**
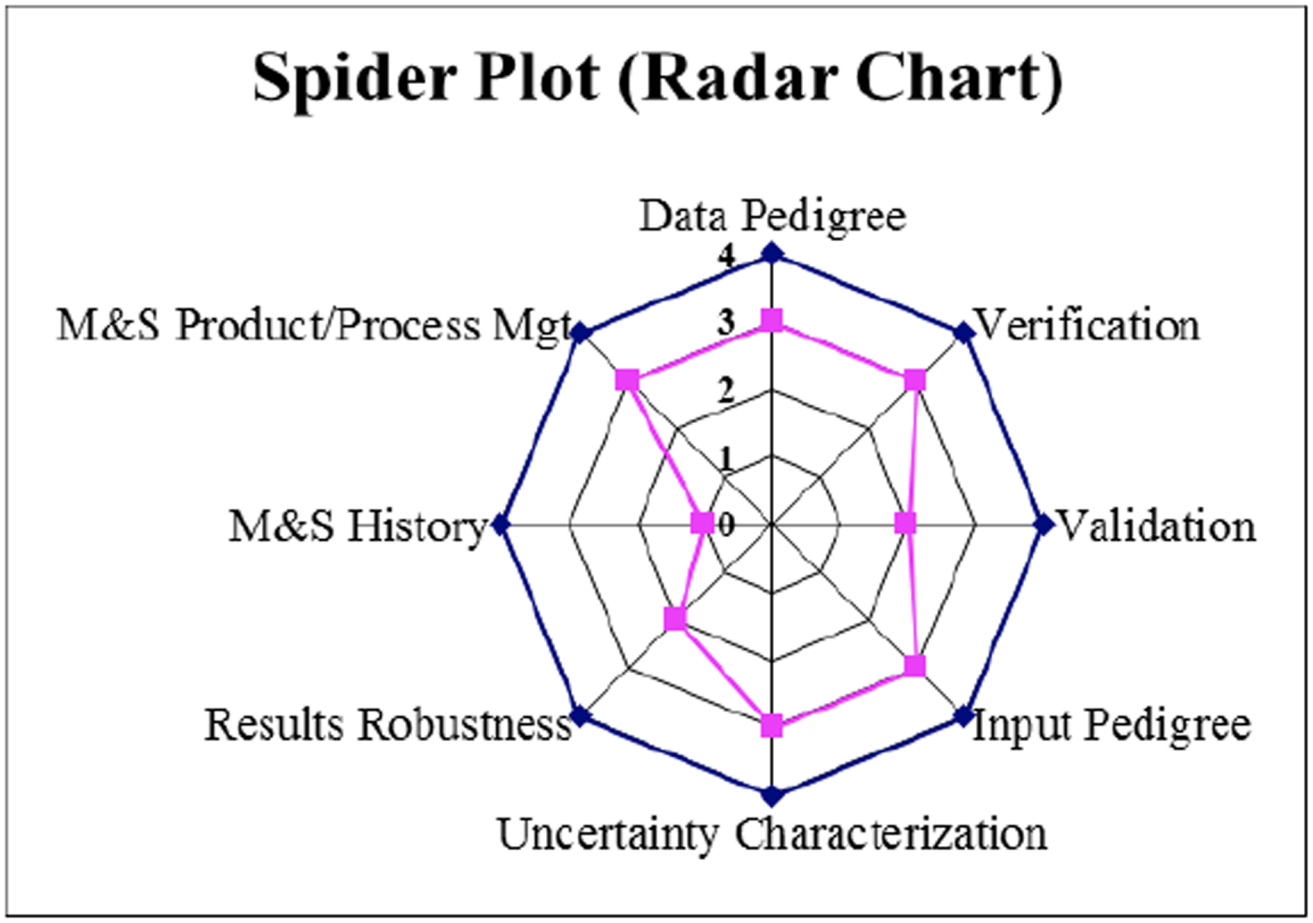
Spider plot or radar plot of credibility assessment.

NASA‐STD‐7009A (https://standards.nasa.gov/standard/nasa/nasa-std-7009) is a “Technical Standard published by the National Aeronautics and Space Administration (NASA) to provide uniform engineering and technical requirements for processes, procedures, practices, and methods endorsed as standard for models and simulations developed and used in NASA programs and projects, including requirements for selection, application, and design criteria of an item.” Although this document is more or less intended for the engineering community, a majority of the elements covered (e.g., Data Pedigree, Verification, Validation, Input Pedigree, Uncertainty Characterization, Robustness) are also applicable to space environment/space science model verification and validation endeavors and can serve as a starting point. Figures 6 and 7 are different representations of Credibility Assessment taken from the document. These elements are also important considerations with validations of space environment models, facilitating standardization of the model assessment processes.

## 7 Initial Set of Space Environment Models and Effect Models

To start the validation efforts, the working team has identified an initial set of potential space environment models for each subtopic area. It should be noted that this is intended to be an evolving community effort. Models do not need to be hosted at Community Coordinated Modeling Center (CCMC) to participate. New models or newer versions of existing models with more capabilities are expected/urged to join once ready. All participating models will be documented (with the version control) and archived at CCMC's Metadata Registry (https://kauai.ccmc.gsfc.nasa.gov/CMR/view/metadata), constantly being updated.

Effect models are not the focus except those atmospheric radiation models for aviation. Once an effect model is chosen, it should/will be used as the unifying translation tool to be applied across all space environment models.

### 7.1 Surface Charging

Space environment models of initial focus are the following: Ovation Prime of the CCMC implementation (https://ccmc.gsfc.nasa.gov/models/modelinfo.php?model=Ovation%20Prime) for characterizing aurora (Newell et al., 2010); the Ring current‐Atmosphere interaction Model and Self‐Consistent Magnetic Field (B) (e.g., Jordanova et al., 2010), and its variants (such its coupling with the Space Weather Modeling Framework; e.g., Yu et al., 2016); the Comprehensive Inner‐Magnetosphere Ionosphere Model (Fok et al., 2014), and its variants (Glocer et al., 2011, 2013); and the Inner Magnetosphere Particle Transport and Acceleration Model (e.g., Ganushkina et al., 2015). Yu et al. (2019) showcase the initial progress in surface charging related validation effort.

#### 7.1.1 Spacecraft Charging Models

Known charging codes include the NASCAP‐2k (NASA/Air Force Spacecraft Charging Analyzer Program; Rubin et al., 1980; Davis & Mandell, 2014); SPIS (Spacecraft Plasma Interaction Software; http://dev.spis.org/projects/spine/home/spis), MUSCAT (Multi‐Utility Spacecraft Charging Analysis Tool; e.g., Muranaka et al., 2007; Hosoda et al., 2008), and other small group ones. Other engineering effect codes relevant to surface charging can be found at SPENVIS (the Space ENVironment Information System; https://www.spenvis.oma.be/).

### 7.2 Internal Charging

#### 7.2.1 Environment Models

The models that have high probability of running benchmarks soon include physics‐based models such as the Comprehensive Inner‐Magnetosphere Ionosphere (Fok et al., 2014), Versatile Electron Radiation Belt code (Shprits et al., 2009; Subbotin and Shprits, 2009), DREAM (a data assimilative model, Reeves et al., 2012), the British Antarctic Survey model (Glauert et al., 2014; Horne et al., 2018), and Salammbo (e.g., Beutier et al., 1995; Bourdarie et al., 2005) and empirical models such as CRRESELE (Brautigam & Bell, 1995). Other more orbit‐specific (e.g., GEO) models include the Geosynchronous Radiation–belt Electron Empirical Prediction model (Kellerman et al., 2013), Relativistic Electron Forecast Model (running at the Space Weather Prediction Center; https://www.swpc.noaa.gov/products/relativistic-electron-forecast-model , Baker et al., 1990), the Ukhorskiy model (Ukhorskiy et al., 2004), the model using Nonlinear Autoregressive Moving Average modeling algorithm (https://ccmc.gsfc.nasa.gov/models/modelinfo.php?model=SNB3GEO; Balikhin et al., 2011; Boynton et al., 2013), and the Li et al. model (e.g., Li et al., 2001; http://lasp.colorado.edu/space_weather/xlf3/xlf4.html).

#### 7.2.2 Effect Models

Internal charging codes such as NUMIT (Jun et al., 2008; Kim et al., 2017), DICTAT (Rodgers, 1999), and SHIELDOSE‐2 (Seltzer, 1994) can be used as the translation tool. DICTAT is to be superseded by MCICT (Monte Carlo Internal Charging Tool; Lei et al., 2016).

### 7.3 Total Dose

Since the main contributors for total dose are electrons >100 keV and protons >1 MeV, with the former mostly of trapped electrons in the Earth's radiation belts and the latter mostly of solar origin, the corresponding initial set of environment models are as follows.

#### 7.3.1 Environment Models

The empirical ones for the trapped population include AE8/AP8 (e.g., Sawyer & Vette, 1976; Vampola, 1996; Vette, 1991), AE9/AP9/SPM (Ginet et al., 2013), IGE2006/POLE (Boscher et al., 2003; Sicard‐Piet et al., 2006, 2008), CRESSELE, and CRESSPRO (Gussenhoven et al., 1994). The empirical ones for particles of solar origin are the King (King, 1974) model, JPL‐91 (Feynman et al., 1993), Emission of Solar Protons/Prediction of Solar particle Yields for CHaracterizing Integrated Circuits (ESP/PSYCHIC model, Xapsos et al., 1999, 2000, 2007), and Solar Accumulated and Peak Proton and Heavy Ion Radiation Environment (SAPPHIRE model, Jiggens et al., 2018).

The physics‐based models for the trapped population are the same as those for the internal charging (section 7.2). For the solar population modeling, there is SOLar Particle ENgineering Code (SOLPENCO, Aran et al., 2005, 2006). Other potential SEP models include those participating in the SEP scoreboard (https://ccmc.gsfc.nasa.gov/challenges/sep.php), such as COronal Mass Ejections and Solar Energetic Particles (COMESEP model, Crosby et al., 2012), SEPForecast, Forecasting Solar Particle Events and Flares (FORSPEF model, Anastasiadis et al., 2017; Papaioannou et al., 2018), UMASEP (Núñez, 2011, 2015; Núñez et al., 2017), PREDICCS (http://prediccs.sr.unh.edu/; Schwadron et al., 2010), AER SEP model (Winter et al., 2015), SPRINTS (Engell et al., 2017), and REleASE/High‐Energy Solar Particle Events foRecastIng and Analysis (e.g., Posner et al., 2007; Malandraki and Crosby, 2018).

Empirical (climatological) models are typically used for total dose calculation for a mission, for example, AP9/AE9 for trapped particles and JPL/ESP for solar protons

#### 7.3.2 Effect Models

Effect models include the NOVICE  code (Jordan, 1976), SHIELDOSE‐2 for total ionizing dose calculation, and EQFLUX and MC‐SCREAM (as done in Hands et al., 2018) for displacement damage dose estimate.

### 7.4 Single‐Event Effects

#### 7.4.1 Environment Models

For the trapped protons, we have AP9 (also AP8 still used in some standards), PSB97, and its updated version (based on SAMPEX/PET data; Heynderickx et al., 1999). For SEP models, we have the ESP/PSYCHIC, JPL, MSU (Nymmik, 1999, 2007), and SAPPHIRE models. As mentioned above, there are a variety of models involved in the SEP Scoreboard activities and the SEP Working Team of the International forum (https://ccmc.gsfc.nasa.gov/assessment/topics/helio-sep.php). Commonly used GCR models include the ISO‐15390 GCR model, Badhwar‐O'Neill (Badhwar & O'Neill, 1996; O'Neill et al., 2011), and the Deutsches Zentrum für Luft‐ und Raumfahrt‐‐German Aerospace Center GCR model (Matthiä et al., 2013). Existing models to assess the SEPs and GCRs' access to the near‐Earth region due to magnetic field shielding include the Energetic Solar Heavy Ion Environment Models–Magnetospheric Shielding Model (Lei, 2017) and the Smart and Shea model (e.g., Smart & Shea, 1994, 2001, 2003).

#### 7.4.2 Effect Models

For the SEE rate calculation, the CRÈME96 software package can be used (https://creme.isde.vanderbilt.edu/; Tylka et al., 1997). Other models include those at SPENVIS.

### 7.5 Radiation Effects at Aviation Altitudes

#### 7.5.1 Environment Models

All the models discussed above regarding SEPs and GCRs also apply here.

#### 7.5.2 Effect Models

First steps have been taken in verification of models assessing radiation exposure at aviation altitudes (Meier et al., 2018, this special issue). The participating models are CARI‐7A at Federal Aviation Administration (e.g., Copeland, 2017), PANDOCA (e.g., Matthiä et al., 2014), and NAIRAS (Mertens et al., 2010, 2013). Other possible models include AVIDOS (e.g., Latocha et al., 2009), QARM (e.g., Lei et al., 2006), KREAM (Hwang et al., 2014), EPCARD. Net (the European Program Package for the Calculation of Aviation Route Doses) e.g., Mares et al., 2009; Schraube et al., 2002), and MAIRE (http://www.radmod.co.uk/maire). Additional ones are also mentioned in Tobiska et al. (2015) and Matthiä et al. (2014).

## 8 Initial Progress

The working team has made some initial progress. In the area of surface charging, some preliminary model validation work has been carried out using the identified physical quantity and a corresponding paper is included in this special issue (Yu et al., 2019). Ganushkina et al., 2019 (also in this special issue) presents validation work done with IMPTAM where the HSS‐type binary event analysis metrics, the median symmetric accuracy, and symmetric signed percentage bias were employed. In the area of internal charging, two major events/periods where internal charging anomalies occurred have been selected. Two manuscripts in the area of radiation effects at aviation altitudes have been published as part of this special issue (Meier et al., 2018; Tobiska et al., 2018). In Meier et al. (2018), the mean deviation was used as a metrics for validating models for the assessment of the radiation exposure at aviation altitudes. For total dose effects, due to its long‐term and accumulative nature, the team has decided to start with how changes in orbit, such as electric orbit raising (usually taking about six months), affect total dose a satellite receives during the duration. In comparison to the other subtopic areas, total dose has some unique aspects in that it is a climatological quantity, not so much a space weather quantity. Total dose estimate for a mission uses a long‐term average environment, not the worst‐case environment. Quantities that are needed for computing total dose include trapped electron and proton fluence spectra and SEP fluence spectra for the duration of a mission. For single‐event effects, we will start with assessment of rigidity cutoff models. Presentations and relevant documents can be found in our Google drive (https://drive.google.com/drive/folders/0Bxc9VBElGQoga2JxRVkta1ZIVXM). In general, the focus team has recognized the importance of energy spectra in leading to a quantitative estimate of engineering impacts discussed in this paper.

## 9 Summary and Future Outlook

With recognition of the complexity (needs knowledge of environment, shielding characteristics, device effects/response, and so on) involved in assessing how space environment affects space assets (both technology and humans), we mainly focus on performance of space environment models but with potential impacts in mind. The quantities chosen for validation have impact bearing and can be qualitatively translated into impact information. Besides calling the community's attention to this rather new type of validation, we hope the quantities identified in Table 1 can serve as a starting point (eventually leading to definition of the standard) in tracking space environment models' usefulness and performance in space weather operations.

**Figure 8:**
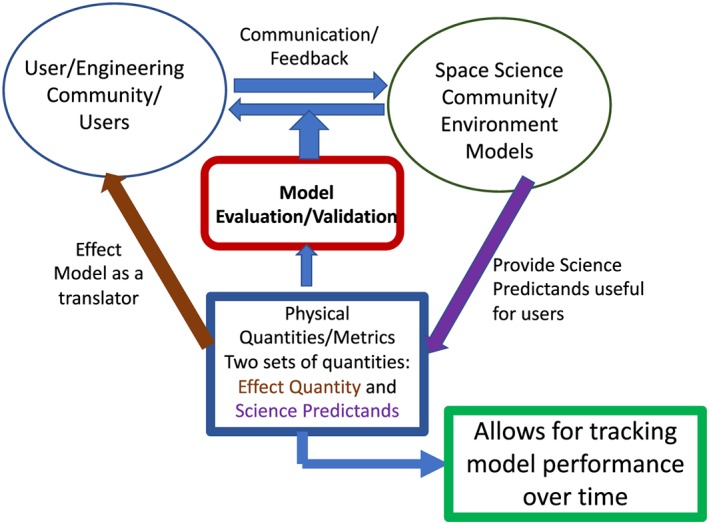
The importance of the model validation efforts with two sets of physical quantities.

Figure 8 summarizes the goal of the effort and puts its importance in a global context in terms of bridging different communities (users in the diagram bears a more general meaning) together. Such effort is indispensable in the research to operations and operations to research arena. Spurring from such initiative, CCMC is building a model inventory (through its Metadata Registry as mentioned above) where specifics of space environment models are documented, such as the version, input, output, language, running platforms, usage/capability, and caveats. Such validation efforts are expected to be archived, either linked to the model inventory or be part of the Metadata Registry, with the ultimate goal of tracking model performance over time for the benefit of different types of users.
